# Marital Status and Prognostic Nomogram for Bladder Cancer With Distant Metastasis: A SEER-Based Study

**DOI:** 10.3389/fonc.2020.586458

**Published:** 2020-10-27

**Authors:** Liangjun Tao, Xinyuan Pan, Lixiang Zhang, Jiawei Wang, Zican Zhang, Li Zhang, Chaozhao Liang

**Affiliations:** ^1^ Department of Urology, The First Affiliated Hospital of Anhui Medical University, Institute of Urology and Anhui Province Key Laboratory of Genitourinary Diseases, Anhui Medical University, Hefei, China; ^2^ Department of Ophthalmology, The Second People's Hospital of Wuhu, Wuhu, China; ^3^ Department of Gastrointestinal Surgery, The First Affiliated Hospital of Anhui Medical University, Hefei, China; ^4^ Department of Urology, The Second People's Hospital of Wuhu, Wuhu, China; ^5^ Clinical College of Bengbu Medical University, Bengbu, China

**Keywords:** bladder cancer, distant metastasis, marital status, nomogram, prognosis

## Abstract

**Background:**

To investigate the impact of marital status on overall survival (OS) and create a prognostic nomogram predicting OS in distant-metastatic bladder cancer (DMBC) patients.

**Methods:**

The Surveillance, Epidemiology, and End Results (SEER) database was explored to recruit DMBC patients from 2010 to 2015. Kaplan–Meier survival analysis was used to compare survival differences among different marital status. Univariate and multivariate analyses were used to screen for prognostic factors and then constructed the nomogram based on Cox proportional hazard regression models. Calibration plot diagrams and concordance index (C-index) were used to verify the prognostic nomogram.

**Results:**

Kaplan–Meier curves suggested the significant differences of OS among different marital status existed in total (*P* < 0.001), female (*P* = 0.011) and male (*P* = 0.001) DMBC patients, respectively. Multivariate analysis indicated marital status was an independent prognostic factor for OS of DMBC patients. Nomogram showed the contribution of marital status to predicting OS was small. Other independent prognostic factors included age, grade, histology type, surgery of primary site, chemotherapy, and metastasis pattern. By combining seven factors, we constructed a prognostic nomogram for DMBC patients. The C-index of this nomogram for OS prediction was 0.722 (95% CI 0.712–0.732). The calibration curves showed perfect consistency between observed and predictive survival.

**Conclusions:**

Marital status was an independent prognostic factor for OS of DMBC patients, but its contribution to predicting OS was small. The prognostic nomogram will provide an individualized evaluation of OS and guidance for suitable treatments in DMBC patients.

## Introduction

Bladder cancer (BC) is the 10th most common cause of cancer and the 13th leading cause of cancer death in the world, with an estimated 549,000 new cases and 200,000 deaths in 2018 (1, 2). BC is approximately four times more common in men than in women and is a disease of the elderly, with 80% of BC patients are over 65 years in the US (3). Marital status has been shown to affect the natural history of many diseases, including several cancers (4). Married patients have improved survival in gallbladder cancer (5), colorectal cancer (6), prostate cancer (7), breast cancer (8), head and neck cancer (9), and so on. Undoubtedly, many studies have investigated the impact of marital status on survival of BC patients (4, 10–13). Klaassen et al. (14) reported that female, black, and unmarried patients are more predisposed to have metastatic BC. However, As far as we know, there are few studies to explore the effect of marital status on survival and evaluate the magnitude of this effect in distant-metastatic bladder cancer (DMBC) patients.

About 10–15% of BC patients already have metastasis at initial diagnosis and 15–30% high-grade BC will eventually progress to advanced disease and lead to poor prognosis (15). The DMBC is mainly hematogenous dissemination, which usually results in metastasis to the liver, lung, bone, and adrenal gland. Once distant metastatic disease has developed, then BC is conventionally viewed as incurable (16, 17). Median survival of DMBC patients is 3–6 months without treatment and approximately 1 year with treatment (18). Thus, it is imperative to construct an exact model to evaluate the prognosis of DMBC patients.

Nomogram is a visible and reliable statistical prediction tool, in which several important factors different from pathological variables, such as age, gender, marital status, race and treatment, are also used to predict the prognosis (19). Thus, we can obtain the probability of personal survival outcomes and direct decisions on treatment by the prognostic nomogram. Some nomograms have been constructed for predicting the survival of BC patients (19–22). Previous studies have also created nomograms to predict the prognosis of metastatic BC patients who received platinum-based chemotherapy and provided reference for the individualized chemotherapy (23, 24). However, to our knowledge, there is no study to perform a prognostic nomogram for the prediction of overall survival (OS) of all the DMBC patients, no matter what treatment they received.

In this study, we exploited data from the Surveillance, Epidemiology, and End Results (SEER) database of BC patients from 2010 to 2015 to analyze the impact of marital status on OS of DMBC patients and evaluate the magnitude of this impact. Moreover, we do our best to create a prognostic nomogram predicting accurate and individualized OS of DMBC patients and evaluate suitable therapeutic modalities.

## Patients and Methods

### Data Source and Patient Selection

The current study data were extracted from the SEER-18 registry of the United States (US) national cancer institute. The SEER database is the largest publicly available cancer dataset. It is a population-based cancer registry across several disparate geographic regions and revised database covering approximately 25% of cancer patients within the United States (25). The SEER*Stat software Version 8.3.5 was utilized to achieve this. To select eligible patients, the search was restricted to cases with the diagnosis of BC from 2010 to 2015. The search was also restricted to cases with distant metastasis at the time of diagnosis (M1 disease by AJCC 7th edition TNM system). The exclusion criteria in our study were as follows: (a) unknown metastatic site; (b) unknown marital status; (c) unknown race; (d) unknown surgery of primary site; (e) unknown chemotherapy; (f) unknown radiotherapy and (g) unknown survival time.

### Data Collection and End Point

The variables from the selected cohorts included: gender, race, age at diagnosis, marital status, histology type, grade, distant metastatic site, surgery of primary site, surgery of lymph node, chemotherapy, radiotherapy, survival months, and vital status. The main end point was OS according to data in the SEER database. OS was defined as the time from diagnosis till death due to any reason.

We divided age into six subgroups: <40, 40–49, 50–59, 60–69, 70–79, and ≥80 years. Metastasis pattern was also divided into six subgroups: bone only, lung only, liver only, brain only, multiple sites, and others. Marital status was divided into four subgroups: married, single, divorced/separated, and widowed. Based on the ICD-O-3, we divided the histology type into transitional cell carcinoma (TCC) (8120/3: transitional cell carcinoma, NOS; 8122/3: transitional cell carcinoma, spindle cell; 8131/3: transitional cell carcinoma, micropapillary), 8130/3:papillary transitional cell carcinoma (PTCC) and others (8020/3: carcinoma, undifferentiated, NOS; 8031/3: giant cell carcinoma; 8082/3: lymphoepithelial carcinoma). Surgery of primary site was divided into three subgroups: no surgery, non-complete cystectomy (local tumor excision; partial cystectomy), and complete cystectomy (complete cystectomy; pelvic exenteration; radical cystectomy).

### Statistical Methods

Student’s t test, Pearson’s chi-square tests, and Fisher’s exact tests were performed for continuous variables and categorical variables. Continuous variables were presented as the mean ± SD. Categorical variables were shown as frequencies and their proportions. Survival estimation and comparison among different variables were performed using Kaplan-Meier analysis and the parameters included mean survival time, median survival time as well as 95% confidence interval (95% CI). The log-rank test was used to compare the significance of the survival curves. Variables determined to be significant in the univariate and multivariate Cox proportional hazards regression analyses were used to generate nomogram to predict 1-, 2-, and 3-year OS. The parameters of Cox proportional hazards regression analysis included hazard ratios (HR) and corresponding 95% CI. Harrell’s concordance-index (C-index) was applied to evaluate the performances of the prognostic nomograms. Consistency between the predicted probability and the observed probability were assessed using calibration curves of the nomogram. Statistical significance was set at two-sided P <0.05. All of the statistical analyses were performed using SPSS software (version 22.0) and R software (version 3.4.3).

## Results

### Baseline Characteristics

A total of 2,715 eligible DMBC patients from 2010 to 2015 were recruited in our study cohort through the SEER database. Our study recruited 750 female (27.6%) and 1,965 male (72.4%). The average age of the whole group was 71.11 ± 11.65 years. Among all patients, 347 (12.8%) patients were divorced/separated, 480 (17.7%) patients were widowed, 457 (16.8%) patients were single, and 1,431 (52.7%) patients were married. As the **Table 1** shown, gender (*P* < 0.001), race (*P* < 0.001), age at diagnosis (*P* < 0.001), grade (*P* = 0.03), surgery of lymph node (*P* = 0.011), and chemotherapy (*P* < 0.001) were all factors that were significantly different among marital status.

**Table 1:** Baseline characteristics of different marital status and all whole cohort.

Variable	Divorced/Separated n (%)	Widowed n (%)	Single n (%)	Married n (%)	P-value	All marital status n (%)
Total	347 (12.8%)	480 (17.7%)	457 (16.8%)	1,431 (52.7%)		2,175 (100%)
Gender					<0.001	
Female	112 (14.9%)	239 (31.9%)	118 (15.7%)	281 (37.5%)		750 (27.6%)
Male	235 (12.0%)	241 (12.3%)	339 (17.3%)	1,150 (58.5%)		1,965 (72.4%)
Race					<0.001	
Other	10 (8.1%)	25 (20.3%)	15 (12.2%)	73 (59.3%)		123 (4.5%)
Black	34 (12.8%)	44 (16.6%)	84 (31.7%)	103 (38.9%)		265 (9.8%)
White	303 (13.0%)	411 (17.7%)	358 (15.4%)	1,255 (53.9%)		2,327 (85.7%)
Age at diagnosis (years)					<0.001	
<40	0 (0.0%)	0 (0.0%)	8 (72.7%)	3 (27.3%)		11 (0.4%)
40–49	12 (14.3%)	1 (1.2%)	34 (40.5%)	37 (44.0%)		84 (3.1%)
50–59	64 (17.3%)	8 (2.2%)	127 (34.2%)	172 (46.4%)		371 (13.7%)
60–69	122 (18.1%)	48 (7.1%)	132 (19.6%)	373 (55.3%)		675 (24.9%)
70–79	100 (11.7%)	157 (18.3%)	91 (10.6%)	508 (59.3%)		856 (31.5%)
≥80	49 (6.8%)	266 (37.0%)	65 (9.1%)	338 (47.1%)		718 (26.4%)
Grade					0.03	
Unknown	55 (12.2%)	86 (19.1%)	89 (19.7%)	221 (49.0%)		451 (16.6%)
Low (grade I–II)	4 (5.1%)	22 (27.8%)	13 (16.5%)	40 (50.6%)		79 (2.9%)
High (grade III– IV)	288 (13.2%)	372 (17.0%)	355 (16.2%)	1,170 (53.5%)		2,185 (80.5%)
Histology type					0.295	
Others	0 (0.0%)	1 (12.5%)	3 (37.5%)	4 (50.0%)		8 (0.3%)
PTCC	92 (11.8%)	155 (19.9%)	131 (16.8%)	400 (51.4%)		778 (28.7%)
TCC	255 (13.2%)	324 (16.8%)	323 (16.7%)	1,027 (53.2%)		1,929 (71.0%)
Surgery of primary site					0.097	
No	67 (12.5%)	96 (17.9%)	106 (19.7%)	268 (49.9%)		537 (19.8%)
Non-complete cystectomy	254 (13.0%)	356 (18.2%)	318 (16.2%)	1,029 (52.6%)		1,957 (72.1%)
Complete cystectomy	26 (11.8%)	28 (12.7%)	33 (14.9%)	134 (60.6%)		221 (8.1%)
Surgery of lymph node					0.011	
No	317 (12.8%)	452 (18.2%)	426 (17.2%)	1,285 (51.8%)		2,480 (91.3%)
Yes	30 (12.8%)	28 (11.9%)	31 (13.2%)	146 (62.1%)		235 (8.7%)
Radiotherapy					0.197	
No	262 (12.3%)	370 (17.3%)	359 (16.8%)	1,147 (53.6%)		2,138 (78.7%)
Yes	85 (14.7%)	110 (19.1%)	98 (17.0%)	284 (49.2%)		577 (21.3%)
Chemotherapy					<0.001	
No	171 (12.6%)	320 (23.6%)	220 (16.2%)	644 (47.5%)		1,355 (49.9%)
Yes	176 (12.9%)	160 (11.8%)	237 (17.4%)	787 (57.9%)		1,360 (50.1%)
Metastasis pattern					0.071	
Bone only	88 (14.1%)	104 (16.7%)	106 (17.0%)	326 (52.2%)		624 (23.0%)
Lung only	62 (12.1%)	95 (18.5%)	91 (17.7%)	265 (51.7%)		513 (18.9%)
Liver only	21 (9.0%)	59 (25.2%)	43 (18.4%)	111 (47.4%)		234 (8.6%)
Brain only	2 (7.4%)	3 (11.1%)	6 (22.2%)	16 (59.3%)		27 (1.0%)
Multiple sites	77 (14.6%)	78 (14.8%)	96 (18.2%)	277 (52.5%)		528 (19.4%)
Other	97 (12.3%)	141 (17.9%)	115 (14.6%)	436 (55.3%)		789 (29.1%)

PTCC, papillary transitional cell carcinoma; TCC, transitional cell carcinoma.

### Kaplan–Meier Survival Analysis for Different Marital Status

**Figure 1:**
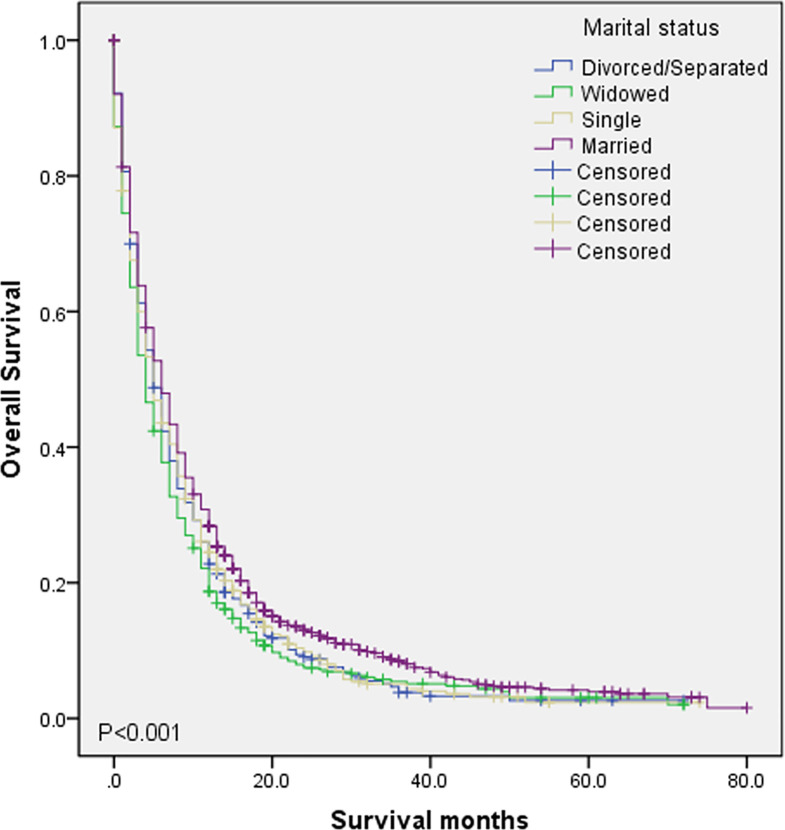
Kaplan–Meier survival analysis of OS among different marital status in DMBC patients (*P* < 0.001).

**Figure 2:**
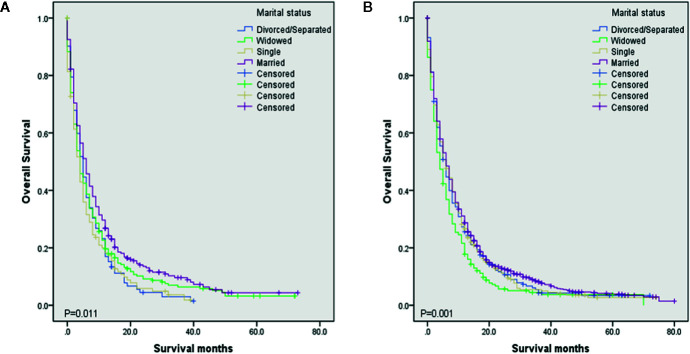
Kaplan–Meier survival analysis of OS among different marital status in different gender. **(A)** OS among different marital status in female DMBC patients (*P* = 0.011). **(B)** OS among different marital status in male DMBC patients (*P* = 0.001).

**Figure 3:**
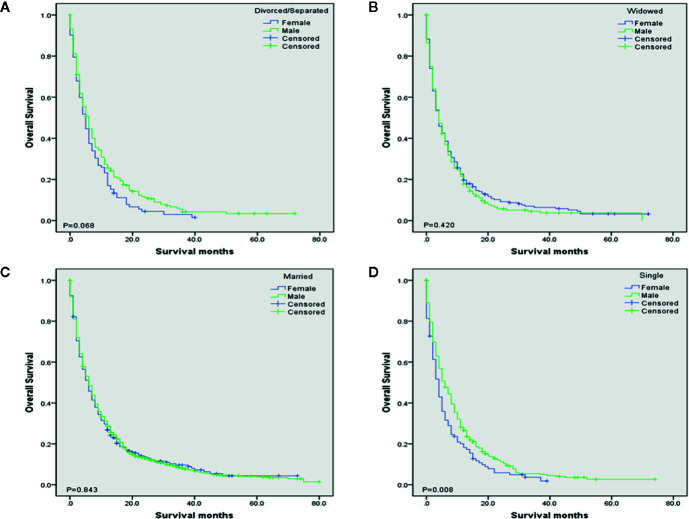
Kaplan–Meier survival analysis of each marital status among different gender in DMBC patients. **(A)** OS of divorced/separated patients between female and male. (*P* = 0.068). **(B)** OS of widowed patients between female and male. (*P* = 0.420). **(C)** OS of married patients between female and male. (*P* = 0.843). **(D)** OS of single patients between female and male. (*P* = 0.008).

To evaluate the impact of different marital status on OS of DMBC patients, we performed Kaplan–Meier survival analysis in total patients. As the **Figure 1** shown, there were significant differences of OS among different marital status (*P* < 0.001). The survival was highest for married patients (median OS = 6, 95%CI = 5.454–6.546), followed by divorced/separated patients (median OS = 5, 95%CI = 4.163–5.837) and single patients (median OS = 5, 95%CI = 4.160–5.840), with the worst survival in widowed patients (median OS = 4, 95%CI = 3.366–4.634). In order to determine whether the effect of marital status on OS was associated with gender, we also performed Kaplan–Meier survival analysis in female and male DMBC patients, respectively. As the **Figure 2A** shown, for female patients, there were significant differences of OS among different marital status (*P* = 0.011) and married patients had the highest survival (median OS = 6, 95%CI = 4.834–7.166). As the **Figure 2B** shown, for male patients, there were also dramatic differences of OS among different marital status (*P* = 0.001) and widowed patients had the worst survival (median OS = 4, 95%CI = 3.128–4.872). Furthermore, we performed Kaplan–Meier survival analysis of each marital status among different gender in DMBC patients. As the **Figure 3** shown, there were no statistically significant differences of OS between female and male in divorced/separated patients (*P* = 0.068, **Figure 3A**), widowed patients (*P* = 0.420, **Figure 3B**), and married patients (*P* = 0.843, **Figure 3C**), respectively. However, in single patients, male presented with better survival, compared with female (*P* = 0.008, **Figure 3D**).

### Prognostic Factors of DMBC Patients

Univariate analysis of OS was shown in **Table 2**. The result showed that gender, age at diagnosis, marital status, grade, histology type, surgery of primary site, surgery of lymph node, chemotherapy, and metastasis pattern were significant prognostic factors. The variables in univariate analysis with a *P*-value of less than 0.05 were included in multivariate analysis. The results indicated that age at diagnosis, marital status, grade, histology type, surgery of primary site, chemotherapy, and metastasis pattern were independent prognostic factors for OS (**Table 3**).

**Table 2:** Univariate analysis of DMBC patients.

Variables	HR (95% CI)	*p*-value
**Statistically significant factors**		
Gender (male vs. female)	1.110 (1.016–1.212)	0.020
Age at diagnosis (years)		
≥80 vs. <40	0.439 (0.227–0.847)	0.014
≥80 vs. 40–49	0.543 (0.424–0.696)	<0.001
≥80 vs. 50–59	0.655 (0.574–0.747)	<0.001
≥80 vs. 60–69	0.666 (0.597–0.744)	<0.001
≥80 vs. 70–79	0.766 (0.692–0.849)	<0.001
Marital status at diagnosis		
Married vs. divorced/separated	1.135 (1.005–1.282)	0.042
Married vs. widowed	1.254 (1.127–1.397)	<0.001
Married vs. single	1.135 (1.017–1.268)	0.024
Grade		
High (III–IV) vs. unknown	1.182 (1.063–1.314)	0.002
High (III–IV) vs. low (I–II)	0.909 (0.716–1.153)	0.431
Histology		
TCC vs. others	1.143 (0.571–2.289)	0.706
TCC vs. PTCC	0.765 (0.700–0.835)	<0.001
Surgery of primary site		
Complete cystectomy vs. no	2.184 (1.839–2.594)	<0.001
Complete cystectomy vs. non-complete cystectomy	1.668 (1.428–1.947)	<0.001
Surgery of lymph node (yes vs. no)	1.686 (1.454–1.956)	<0.001
Chemotherapy (yes vs. no)	2.507 (2.312–2.718)	<0.001
Metastasis pattern		
Bone only vs. lung only	0.895 (0.793–1.010)	0.073
Bone only vs. liver only	1.210 (1.036–1.414)	0.016
Bone only vs. brain only	1.119 (0.755–1.657)	0.575
Bone only vs. multiple sites	1.519 (1.349–1.712)	<0.001
Bone only vs. others	0.726 (0.649–0.811)	<0.001
**Statistically non-significant factors**		
Race		
White vs. others	0.937 (0.773–1.136)	0.505
White vs. black	1.062 (0.931–1.212)	0.371
Radiotherapy (yes vs. no)	0.979 (0.890–1.077)	0.667

PTCC, papillary transitional cell carcinoma; TCC, transitional cell carcinoma.

**Table 3:** Multivariate analysis of DMBC patients.

Variables	HR (95% CI)	*p*-value
**Statistically significant factors**		
Age at diagnosis (years)		
≥80 vs. <40	0.722 (0.371–1.404)	0.337
≥80 vs. 40–49	0.664 (0.514–0.857)	0.002
≥80 vs. 50–59	0.822 (0.712–0.948)	0.007
≥80 vs. 60–69	0.872 (0.773–0.984)	0.026
≥80 vs. 70–79	0.943 (0.846–1.051)	0.288
Marital status at diagnosis		
Married vs. divorced/separated	1.094 (0.966–1.239)	0.155
Married vs. widowed	0.987 (0.878–1.110)	0.828
Married vs. single	1.153 (1.029–1.292)	0.014
Grade		
High (III–IV) vs. unknown	0.897 (0.795–1.013)	0.080
High (III–IV) vs. low (I–II)	0.715 (0.561–0.910)	0.006
Histology type		
TCC vs. others	0.894 (0.443–1.804)	0.755
TCC vs. PTCC	0.771 (0.705–0.844)	<0.001
Surgery of primary site		
Complete cystectomy vs. no	1.457 (1.071–1.983)	0.016
Complete cystectomy vs. non-complete cystectomy	1.204 (0.903–1.605)	0.207
Chemotherapy (yes vs. no)	2.423 (2.224–2.640)	<0.001
Metastasis pattern		
Bone only vs. lung only	0.884 (0.781–1.000)	0.050
Bone only vs. liver only	1.110 (0.948–1.299)	0.195
Bone only vs. brain only	1.408 (0.949–2.089)	0.089
Bone only vs. multiple sites	1.561 (1.384–1.760)	<0.001
Bone only vs. others	0.804 (0.718–0.900)	<0.001
**Statistically non-significant factors**		
Gender (male vs. female)	1.046 (0.954–1.147)	0.334
Surgery of lymph node (yes vs. no)	1.260 (0.955–1.664)	0.103

PTCC, papillary transitional cell carcinoma; TCC, transitional cell carcinoma.

### Prognostic Nomogram for OS

**Figure 4:**
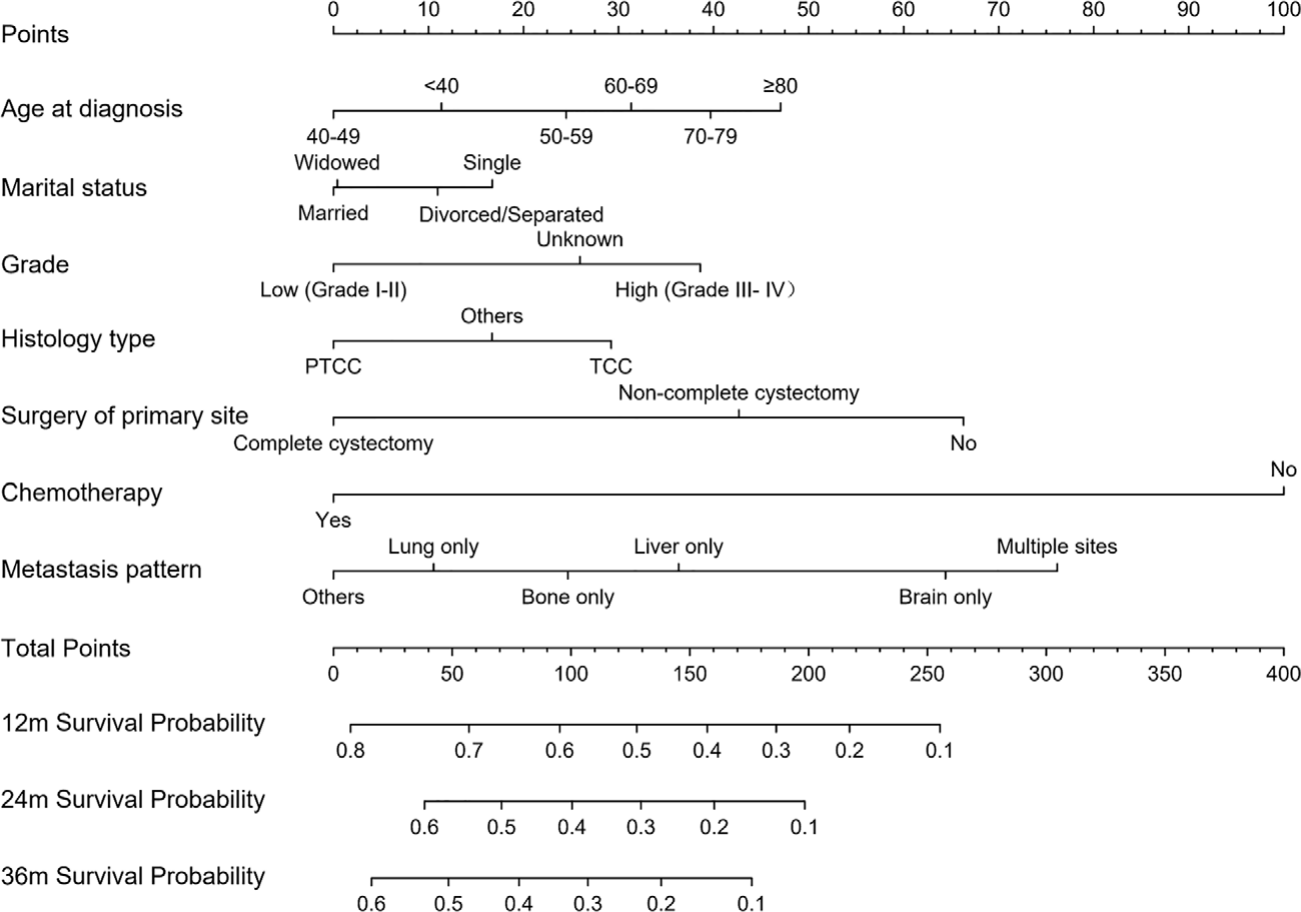
Nomogram for predicting OS of DMBC patients. (PTCC, papillary transitional cell carcinoma; TCC, transitional cell carcinoma).

The 1-, 2-, and 3-year OS of DMBC patients were predicted by constructing a nomogram based on Cox regression models (**Figure 4**). Each subgroup variable was assigned a corresponding score for the construction of this nomogram. A score system was used to assign a score of 0 to 100 for each subgroup variable according to its contribution. These scores were added across enrolled variables to generate total scores on the bottom scales, which were then transformed to predict the corresponding OS. The nomogram demonstrated that chemotherapy was the largest contributor to prognosis, followed by metastasis pattern and surgery of primary site. Age at diagnosis, grade, histology type, and marital status also showed a moderate effect on OS. The nomogram scoring system was shown in **Table 4**.

**Table 4:** Nomogram scoring system.

Variables	Points	Variables	Points	Variables	Points
Age at diagnosis (years)		Metastasis pattern		Grade	
<40	11	Bone only	25	Unknown	26
40–49	0	Lung only	11	Low (I–II)	0
50–59	24	Liver only	36	High (III–IV)	39
60–69	31	Brain only	64	Chemotherapy	
70–79	40	Others	0	No	100
≥80	47	Multiple sites	76	Yes	0
Marital status		Histology type		Surgery of primary site	
Divorced/separated	11	Others	17	No	66
Widowed	0	PTCC	0	Non-complete cystectomy	43
Single	17	TCC	29	Complete cystectomy	0
Married	0				
**1-Year OS probability**	**Points**	**2-Year OS probability**	**Points**	**3-Year OS probability**	**Points**
0.8	7	0.6	38	0.6	16
0.7	57	0.5	71	0.5	48
0.6	95	0.4	100	0.4	78
0.5	128	0.3	129	0.3	107
0.4	157	0.2	160	0.2	138
0.3	186	0.1	198	0.1	176
0.2	217				
0.1	255				

PTCC, papillary transitional cell carcinoma; TCC, transitional cell carcinoma.

### Validation of the Nomogram

**Figure 5:**
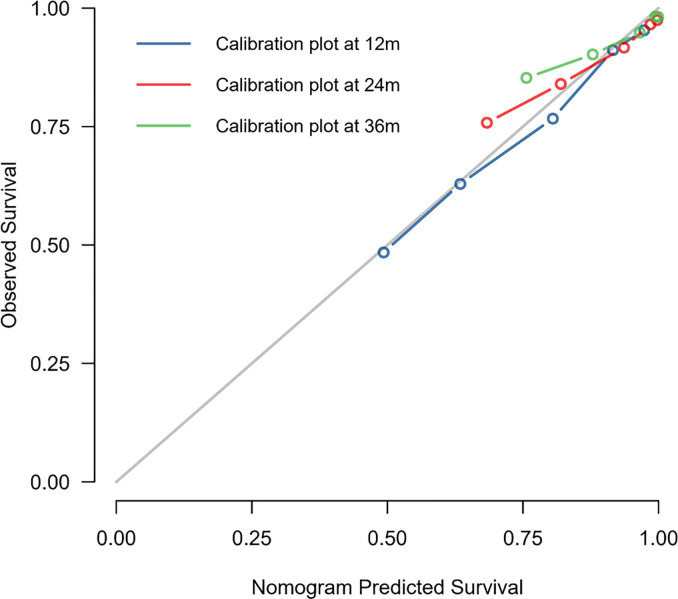
Calibration curves of the prognostic nomogram for 1-, 2-, and 3-year OS in DMBC patients.

The C-index of this nomogram for OS prediction was 0.722 (95% CI 0.712–0.732), which was greater than 0.7, suggesting the suitability of our nomogram for DMBC patients. In addition, the calibration curve was used to validate the model’s ability for predicting the 1-, 2-, and 3-year OS of DMBC patients. As the **Figure 5** shown, a perfect correlation between nomogram prediction and observed outcomes demonstrating great reliability of the nomogram.

### Kaplan–Meier Curves for Nomogram

**Figure 6:**
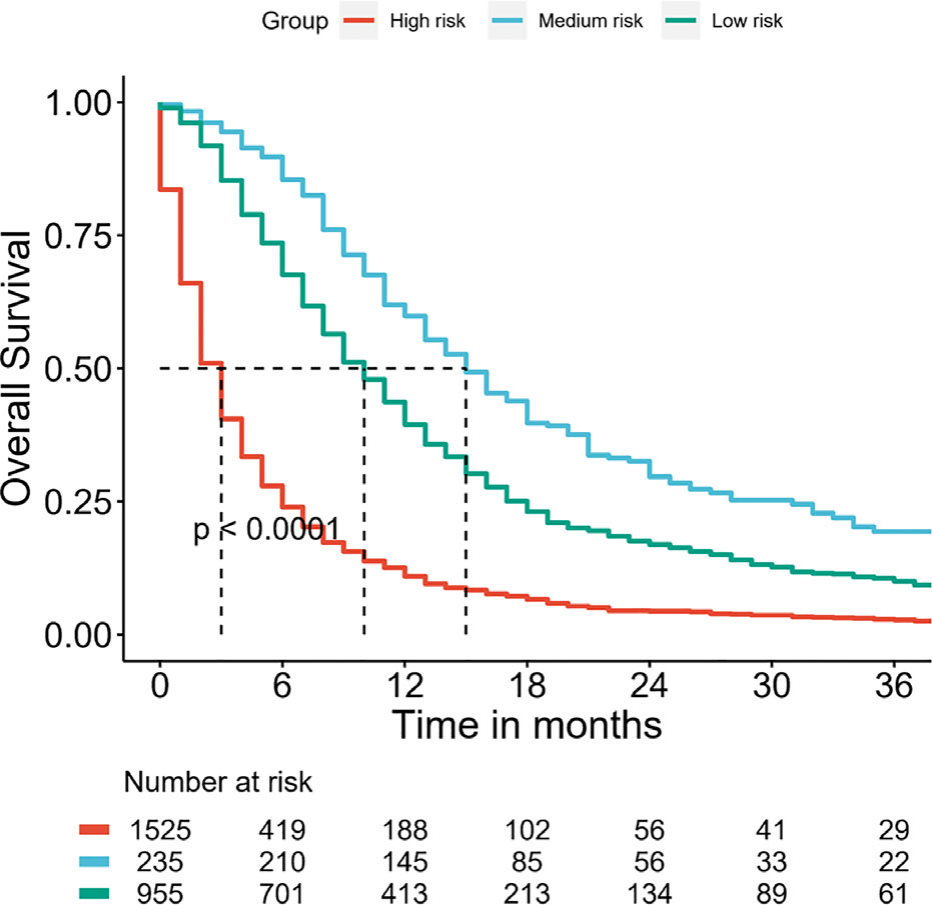
Survival curves stratified by the score calculated by the nomogram in DMBC patients. (low risk: ≤124; medium risk: 125–199; and high risk: ≥200).

Furthermore, the DMBC patients were divided into three subgroups according to the total points of the nomogram: low risk: ≤124, medium risk: 125–199 and high risk: ≥200. As the **Figure 6** shown, the Kaplan–Meier curves revealed an excellent prediction results in the prognostic nomogram.

## Discussion

Marital status has been confirmed to have a significant impact on survival in many cancers including BC. Gore et al. (26) suggested that married BC patients had a 20% increased survival in comparison with single patients and 44% increased survival in comparison with widowed patients after radical cystectomy (RC). Seo et al. (11) reported improved survival in married patients with non–muscle-invasive bladder ancer (NMIBC). Sammon et al. (4) showed being married is protective factor for both men and women in BC patients after RC. Nelles et al. (10) found widowed male patients with BC had an increased risk of death. Although klapheke et al. (12) suggested married metastatic BC patients had a good prognosis, he did not evaluated the contribution of marital status to predicting prognosis of metastatic BC patients.

In this study, we comprehensively explored the effect of marital status on OS of DMBC patients and evaluated the magnitude of this effect by our nomogram. We observed that there were significant differences of OS among different marital status in total, female and male DMBC patients, respectively. Married DMBC patients presented with improved survival both for female and male. Some proposed mechanisms can be used to explain the association between cancer survival and marital status. Patients who are married may obtain increased financial resources, may experience improved social support, may enjoy higher quality of life, may receive better treatment than patients who are unmarried (4). Klapheke et al. (12) reported unmarried patients were less likely to have chemotherapy in metastatic BC patients. In this study, we also observed the proportion of married patients (787/1,360, 57.9%) was higher than unmarried patients (573/1,360, 42.1%) in received chemotherapy DMBC patients, which suggested married patients were more likely to have chemotherapy.

Multivariate analysis indicated marital status was an independent prognostic factor for OS of DMBC patients. However, in univariate analysis, compared with married, the HR of divorced/separated, widowed and single was 1.135 (1.005–1.282, *P* = 0.042), 1.254 (1.127–1.397, *P* < 0.001) and 1.135 (1.017–1.268, *P* = 0.024), respectively; In multivariate analysis, compared with married, the HR of divorced/separated, widowed, and single was 1.094 (0.966–1.239, *P* = 0.155), 0.987 (0.878–1.110, *P* = 0.828) and 1.153 (1.029–1.292, *P* = 0.014), respectively. Thus, marital status as a predictor for OS of DMBC patients was not stable and susceptible to other factors. Sammon et al. (4) also reported the effect of marital status on outcomes of BC patients to be variable, depending on gender and the outcome addressed. Furthermore, our nomogram showed marital status presented with small contribution to predicting OS of DMBC patients, which could also be attributed to marital status being susceptible to some other factors, although it was an independent prognostic factor.

BC is prone to recurrence and metastasis, once distant metastatic disease has developed, then bladder cancer is conventionally viewed as incurable (16, 17). Recently, a number of nomograms, a convenient and reliable statistical prediction tool, have been established for predicting the prognosis of BC patients. However, to our knowledge, few studies focus on the prognostic nomogram for DMBC patients. The current SEER-based study attempted firstly to create prognostic nomogram to evaluate the probability of 1-, 2-, and 3-year OS and to make a highly reliable model of DMBC patients. Multivariate analysis suggested age at diagnosis, marital status, grade, histology type, surgery of primary site, chemotherapy, and metastasis pattern were independent prognostic factors for OS. Thus, we constructed a nomogram of these predictors. The C-index was 0.722 (95% CI 0.712–0.732) and the calibration curves showed a perfect consistency between the nomogram prediction and observed outcomes, suggesting great reliability of the nomogram predicting prognosis for DMBC patients. Our results also showed that this model can well divide patients into high-risk, medium-risk and low-risk groups with significant differences in OS.

This novel nomogram included seven clinical and pathological variables to optimize the prediction of OS for DMBC patients. In our nomogram, chemotherapy was the largest contributor to prognosis. Although targeted therapy and immune- otherapy are promising, chemotherapy still presented with maximal survival benefit for DMBC patients. The 2018 NCCN guidelines also suggested platinum-based chemotherapy has been standard of care in patients with metastatic disease, with an OS of 9 to 15 months (27). A previous study suggested that different distant-metastatic site and multiple sites metastasis were independent prognostic factors for OS in metastatic BC patients (28), which was consistent with our findings. As shown in nomogram, metastasis pattern was the second large contributor to prognosis. Compared with the most common bone only metastasis, multiple sites metastasis had the worst survival.

The effect of surgery of primary site on the prognosis of metastatic BC is still controversial. Alfred et al. (29) thought the role of surgery in metastatic BC was not yet established with most of the experience being accrued from retrospective uncontrolled studies. Recently, a systematic review showed that cytoreductive radical cystectomy as local treatment has also been explored in patients with metastatic disease, but its benefits remain to be assessed (30). However, Herr et al. (31) indicated that surgery of the primary BC might contribute to long-term disease-free survival in selected patients. Dong et al. (28) also suggested surgeries, including radical cystectomy and metastasectomy, might still lead to survival benefits for highly selected patients. A recent study indicated that surgery of the primary tumor site was associated with improved survival in metastatic BC patients who received standard chemotherapy and this effect disappeared in patients affected by two or more metastatic sites (32). In our nomogram, surgery of primary site was the third large contributor to prognosis. Compared with complete cystectomy, no surgery patients presented with the worst survival. In addition, as shown in nomogram, age at diagnosis, grade and histology type also showed a moderate effect on OS. In DMBC patients, ≥80, high grade, and TCC patients had poorer survival.

In this study, we also tried to investigate the impact of marital status on cancer specific survival (CSS) of DMBC patients and evaluate the magnitude of this impact by creating nomogram. However, only four independent factors were screened out by univariate and multivariate analysis (**Table S1** and **S2**) and marital status was no longer an independent predictor of CSS for DMBC patients. We believe the main reason is that the records of death causes are not detailed and accurate in SEER database, which leads to the high proportion of non-bladder cancer cause of death. In SEER database, the ratio of non-bladder cancer death causes to bladder cancer death causes is about 1:3, which is much higher than the actual situation.

To the best of our knowledge, this is the first SEER-based study investigating the impact of marital status on OS and constructing a prognostic nomogram for OS in DMBC patients. However, several limitations should be considered in our study. First of all, this is a retrospective study from SEER database, so the inherent selection biases may undermine the external validity of this study. Meanwhile, external validation cohorts are needed to confirm the predictive accuracy of the nomogram. Second, the data of metastatic sites and causes of death from this database is incomplete and the follow-up time is not long enough. Third, the information about systemic therapy options and recurrence are not available in the SEER database. Finally, we do not obtain other potential prognostic factors such as smoking status, comorbidities, preoperative serum markers and relevant molecular markers from the SEER database.

In conclusion, there were significant differences of OS among different marital status in total, female and male DMBC patients, respectively. Marital status was an independent prognostic factor for OS, but its contribution to predicting OS was small. Other independent prognostic factors included age at diagnosis, grade, histology type, surgery of primary site, chemotherapy, and metastasis pattern. By combining seven factors, we constructed a prognostic nomogram for DMBC patients. The model will provide an individualized evaluation of OS and guidance for suitable treatments in DMBC patients.

## Data Availability Statement

Publicly available datasets were analyzed in this study. This data can be found here: https://seer.cancer.gov/data/. 

## Author Contributions

LJT and CZL designed the study. XYP provided the databases. LJT, LXZ, JWW, ZCZ, and LZ assembled and analyzed the data. LJT wrote the manuscript. All authors contributed to the article and approved the submitted version.

## Conflict of Interest

The authors declare that the research was conducted in the absence of any commercial or financial relationships that could be construed as a potential conflict of interest.
