# Supplementary material for: Marital Status and Prognostic Nomogram for Bladder Cancer With Distant Metastasis: A SEER-Based Study
Source: Front Oncol. 2020 Oct 27;10:586458. doi: 10.3389/fonc.2020.586458 (PMC7654226; doi:10.3389/fonc.2020.586458)
Supplement: Supplementary file 1 [file Table_1.docx]

**TABLE S1. Univariate analysis of CSS in DMBC patients**

| **Variables** | **HR (95% CI)** | ***p*-value** |
| --- | --- | --- |
| **Statistically significant factors** | | |
| Age at diagnosis (years) |  |  |
| ≥80 vs.＜40 | 0.379 (0.169-0.848) | 0.018 |
| ≥80 vs. 40-49 | 0.502 (0.379-0.665) | < 0.001 |
| ≥80 vs. 50-59 | 0.605 (0.521-0.702) | < 0.001 |
| ≥80 vs. 60-69 | 0.624 (0.549-0.709) | < 0.001 |
| ≥80 vs. 70-79 | 0.732 (0.649-0.825) | < 0.001 |
| Marital status at diagnosis |  |  |
| Married vs. divorced/separated | 1.128 (0.983-1.294) | 0.086 |
| Married vs. widowed | 1.355 (1.194-1.537) | < 0.001 |
| Married vs. single | 1.137 (1.002-1.292) | 0.047 |
| Histology |  |  |
| TCC vs. others | 1.223 (0.458-3.265) | 0.688 |
| TCC vs. PTCC | 0.762 (0.689-0.842) | < 0.001 |
| Surgery of primary site |  |  |
| Complete cystectomy vs. no | 2.009 (1.648-2.449) | < 0.001 |
| Complete cystectomy vs. non-complete cystectomy | 1.702 (1.432-2.024) | < 0.001 |
| Surgery of lymph node (yes vs. no) | 1.697 (1.437-2.005) | < 0.001 |
| Chemotherapy (yes vs. no) | 2.632 (2.397-2.889) | < 0.001 |
| Metastasis pattern |  |  |
| Bone only vs. lung only | 0.897 (0.782-1.029) | 0.121 |
| Bone only vs. liver only | 1.142 (0.949-1.375) | 0.160 |
| Bone only vs. brain only | 1.090 (0.697-1.706) | 0.705 |
| Bone only vs. multiple sites | 1.493 (1.303-1.710) | < 0.001 |
| Bone only vs. others | 0.704 (0.620-0.801) | < 0.001 |
| **Statistically non-significant factors** | | |
| Gender (male vs. female) | 1.093 (0.988-1.210) | 0.084 |
| Grade |  |  |
| High (III-IV) vs. unknown | 1.118 (0.985-1.270) | 0.085 |
| High (III-IV) vs. low (I-II) | 0.918 (0.703-1.199) | 0.531 |
| Race |  |  |
| White vs. others | 0.870 (0.686-1.103) | 0.251 |
| White vs. black | 0.987 (0.842-1.157) | 0.869 |
| Radiotherapy (yes vs. no) | 0.927 (0.831-1.034) | 0.173 |

PTCC: papillary transitional cell carcinoma; TCC: transitional cell carcinoma
