# Supplementary material for: Marital Status and Prognostic Nomogram for Bladder Cancer With Distant Metastasis: A SEER-Based Study
Source: Front Oncol. 2020 Oct 27;10:586458. doi: 10.3389/fonc.2020.586458 (PMC7654226; doi:10.3389/fonc.2020.586458)
Supplement: Supplementary file 2 [file Table_2.docx]

**TABLE S2. Multivariate analysis of CSS in DMBC patients**

| **Variables** | **HR (95% CI)** | ***p*-value** |
| --- | --- | --- |
| **Statistically significant factors** | | |
| Age at diagnosis (years) |  |  |
| ≥80 vs.＜40 | 0.708 (0.313-1.601) | 0.407 |
| ≥80 vs. 40-49 | 0.684 (0.513-0.913) | 0.010 |
| ≥80 vs. 50-59 | 0.803 (0.683-0.944) | 0.008 |
| ≥80 vs. 60-69 | 0.866 (0.753-0.996) | 0.044 |
| ≥80 vs. 70-79 | 0.932 (0.821-1.057) | 0.272 |
| Histology type |  |  |
| TCC vs. others | 0.740 (0.274-1.996) | 0.552 |
| TCC vs. PTCC | 0.731 (0.660-0.810) | < 0.001 |
| Chemotherapy (yes vs. no) | 2.488 (2.254-2.747) | < 0.001 |
| Metastasis pattern |  |  |
| Bone only vs. lung only | 0.882 (0.767-1.013) | 0.076 |
| Bone only vs. liver only | 1.074 (0.891-1.295) | 0.452 |
| Bone only vs. brain only | 1.363 (0.870-2.136) | 0.177 |
| Bone only vs. multiple sites | 1.514 (1.321-1.736) | < 0.001 |
| Bone only vs. others | 0.787 (0.690-0.897) | < 0.001 |
| **Statistically non-significant factors** | | |
| Marital status at diagnosis |  |  |
| Married vs. divorced/separated | 1.100 (0.957-1.265) | 0.180 |
| Married vs. widowed | 1.086 (0.951-1.241) | 0.224 |
| Married vs. single | 1.138 (0.999-1.297) | 0.052 |
| Surgery of primary site |  |  |
| Complete cystectomy vs. no | 1.189 (0.836-1.692) | 0.336 |
| Complete cystectomy vs. non-complete cystectomy | 1.135 (0.812-1.586) | 0.458 |
| Surgery of lymph node (yes vs. no) | 1.323 (0.956-1.832) | 0.091 |

PTCC: papillary transitional cell carcinoma; TCC: transitional cell carcinoma
